# Antibacterial Textile Based on Hydrolyzed Milk Casein

**DOI:** 10.3390/ma14020251

**Published:** 2021-01-06

**Authors:** Kedafi Belkhir, Caroline Pillon, Aurélie Cayla, Christine Campagne

**Affiliations:** 1 GEMTEX—Laboratoire de Génie et Matériaux Textiles, ENSAIT, F-59000 Lille, France; kedafi.belkhir@centralelille.fr (K.B.); aurelie.cayla@ensait.fr (A.C.); 2 Université de Lyon, F-42023 Saint Etienne, France; caroline.pillon@univ-st-etienne.fr; 3 CNRS, UMR5223, Ingénierie des Matériaux Polymères, F-42023 Saint-Etienne, France; 4 Université Jean Monnet de Saint Etienne, F-42023 Saint-Etienne, France

**Keywords:** antibacterial textiles, hydrolyzed casein, filaments, melt spinning, synthetic fibers, polypropylene

## Abstract

Antimicrobial textile structures are developed based on polypropylene (PP) and a natural material, hydrolyzed casein. The casein, from bovine milk, is subjected to acid hydrolysis in aqueous media, then blended into the PP matrix in the melt phase by extrusion. The obtained blend, containing 5 wt.% of hydrolyzed casein, is then processed by a melt spinning process to get multifilaments, leading to the production knitting structures. Thanks to the addition of the hydrolyzed casein, the obtained textile showed a strong antibacterial activity towards both Gram (+) and Gram (−) bacterial strains. The addition of 5 wt.% hydrolyzed casein does not significantly impact the mechanical properties of PP in the dumbbells form, but a small decrease was observed in the tenacity of the filaments. No moisture retention was observed after the addition of hydrolyzed casein, but the rheological behavior was slightly affected. The obtained results can contribute to addressing concerns regarding nonrenewable antibacterial agents used in textile materials, particularly their effects on the environment and human health, by offering antibacterial agents from a biobased and edible substance with high efficiency. They are also promising to respond to issues of wasting dairy products and recycling them, in addition to the advantages of using melt processes.

## 1. Introduction

The inclusion of microbiological properties in textile materials is a current and well-investigated research topic; several works were carried out for this purpose during the last decade [1,2,3,4,5,6,7,8]. The growing interest in this idea is due to two main reasons: firstly, textiles are enabling environments for both the growth and the transmission of microorganisms; this is due to their ability to retain moisture and also to being used at temperatures favoring microbial growth [9,10]. Secondly, they are produced more and more massively, finding applications in all areas (clothing, domestic uses, medical applications, transport, etc.). Different active biocides, such as antimicrobial, fungicidal or antifouling agents, are incorporated in textile materials and their surfaces, to make them bioactive. The most commonly used are metals and metallic salts; for example, silver showed its efficiency when incorporated into textiles in the form of nanoparticles [11,12,13] or in the form of powder and silver salts [14,15]. Copper is also used in different forms and showed antibacterial activity against many bacterial stains [16,17,18]. Other metals are also used, such as zinc [19,20], titanium [21], iron [22], and their oxides. Despite their antimicrobial efficiency, metal-based antimicrobial textiles are subjected to studies discussing their harmful effect on the environment [9,23]. The potential hazards to both human and environmental health of these antimicrobial agents are a critical ongoing subject of research, both during the production, the use, and the end life of textile materials [24,25]. In this context, the use of biobased and renewable antibacterial agents is a relevant alternative to answer these concerns.

More than 20 wt.% of dairy products are lost or wasted during their processing, distribution, or consumption [26]. This waste can be recovered to extract the proteins it contains. Cow’s milk contains about 3.3 wt.% of proteins [27], but this content is much higher in other dairy products, such as yogurts and cheeses. Milk proteins are made of 80% casein and 20% whey [28]. Casein is hydrolysable and the resulting hydrolysates show efficient antibacterial activity against several strains [29,30]. The idea of incorporating hydrolyzed casein, as an antibacterial agent, in textile materials offers two relevant advantages: the first one is the biosourced and renewable aspect, and the second one is the valuation and recovery of dairy wastes.

There are several methods allowing the incorporation of antibacterial entities in polymeric textile materials; the first approach consists of covalently linking the antibacterial agent to the polymer backbone, and thereby the leaching of antibacterial entities is avoided. This kind of textile is obtained by chemically linking the antibacterial agent to polymer chains (post-polymerization modification), either at the surface of the textile material or in its bulk [31,32]. It can also obtained by the polymerization of antibacterial monomers [33,34]. The antibacterial activity can be also introduced to the textile polymeric material during the padding steps, allowing one to coat the fabrics with antibacterial agents-containing binders [35,36], or during the finishing steps of its production process, such as dyeing steps [37]. Another approach consists of melt blending of the antibacterial species with the polymer matrix; this method is effective, inexpensive, environmentally friendly and achievable in simple processes [38,39,40,41,42].

The purpose of this work is to prepare hydrolyzed casein-based antibacterial textile materials. Extrusion and melt spinning methods are used to incorporate the hydrolyzed casein in the polymer matrix of polypropylene. This approach also aims to give an answer to environmental and health issues by using a biosourced, renewable and edible product in melt processes. It also offers an opportunity to usefully recycle dairy waste.

## 2. Experimental

### 2.1. Materials

The casein used was of technical grade and isolated from bovine milk as a white powder. Aqueous 6 mol L^−1^ hydrochloric acid (HCl) solution was used as the hydrolysis medium. Both casein and HCl solution were purchased from Sigma Aldrich, Saint-Quentin Fallavier, France. Polypropylene PPH9069 spinning grade (PP) was purchased from Total France and used as received.

### 2.2. Casein Hydrolysis

Hydrolysis of casein was achieved at an ambient temperature; in a sealed glass bulb equipped with a magnetic stirrer, 12 g of casein was mixed with 160 mL of the HCl aqueous solution. The mixture was stirred for 12 h to achieve the hydrolysis reaction. The obtained solution was then concentrated and the hydrolyzed casein was recovered as a black paste. The paste was subjected to drying under vacuum, at 100 °C, for 12 h before using it in the melt processes.

### 2.3. Size Exclusion Chromatography

A Shimadzu High-Performance Liquid Chromatograph (HPLC) was used to perform size exclusion chromatography (SEC) analyses, with an LC-20AD pump (Liquid Chromatography pump with Automatic solvent Delivery) and an RID-10A (Refractive Index Detector). The eluent flow was set to 0.5 mL min^−1^. The flow crosses two consecutive columns: PL aquagel OH mixed H 8 µm and PL aquagel OH mixed M 8 µm. The two columns are of 300 mm length and 7.5 mm inner diameter. They are compatible with a pH range of 2–10. The first one resolves between 100 and 10,000,000 mol/g, and the second one resolves up to 600,000 g/mol. The eluent was a 0.05 mol L^−1^ NaCl aqueous solution.

### 2.4. Extrusion

A Thermo-Haake co-rotating twin-screw extruder (L/D = 25, where L is the useful flighted length of the screw and D the barrel diameter) was used to prepare blends of PP filled with hydrolyzed casein. The apparatus was purchased from Thermo Fisher Scientific, Waltham, MS, USA. The screws rotate at 100 rpm and move the matter through five heating zones with different temperatures from the feed zone (Z_1_) to the die (Z_5_), Table 1 shows the temperature profile. Once out of the die, the blend was air-cooled and granulated. A masterbatch was first prepared containing 10 wt.% of hydrolyzed casein, then the obtained blend underwent a second extrusion, in the same conditions, in order to dilute the hydrolyzed casein concentration to 5 wt.% by adding the proper amount of PP during feeding (each kg of the masterbatch was blended with 1 kg of neat PP).

### 2.5. Melt Spinning

A SPINBOY I melt spinning machine, from Busschaert Engineering Belgium, was used to prepare multifilament yarns from the extruded blend (PP with 5 wt.% hydrolyzed casein). The blend was introduced to the spinning machine from the feed hopper, and then convoyed by a rotatory screw through six heating zones (z_1_ to z_6_) as in a single-screw extruder L/D = 25. The melt blend was thus pushed to cross two dies with 40 holes in each one (1.2 mm diameter holes), thanks to a volumetric rotatory pump (16 rpm) which ensures a constant flow. The obtained 80 filaments were combined to make a yarn, and wrapped around two heated rolls that rotate at different speeds (*S*_1_ and *S*_2_). The two rolls stretched the yarn, due to the difference in the rotating speed, and the draw ratio *R* is theoretically equal to $\frac{S_{2}}{S_{1}}$. The spinning process parameters are listed in Table 2. The obtained filaments are of 31.5 dTex, and the yarn of 251.9 dTex (1 dTex = 1 deci−Tex = 1 dg/km = 0.1 mg/m is the linear density of a filament or a yarn: it provides the weight, in dg, of 1 km of filament or yarn). The obtained yarn was used to make a knit (Figure S1 in Supplementary Materials).

A5 format textile structures were prepared using a manual knitting machine. The knit fabric had to have a large pore structure for the antibacterial test (agar), so that bacteria can breathe underneath the knit. In fact, the bacteria used in this study multiply in the presence of oxygen—they are thus called aerobic. All samples were knitted in rib 1 × 1 with 69 needles and E8 gauge. For the antibacterial test, the knitting structures were desired for removing the presence of oil and surfactant at the yarns surface. Three steps were required for the desizing; the first step is several cycles in a soxhlet containing petroleum ether for 4 h. Fabrics were dried for at last 12 h, and then underwent a second soxhlet in ethanol for the same time as before. Three cleanings in distilled water under ultrasound (37 Hz) for 20 min released residual products from the sizing. After drying the knits for 12 h, they could be characterized.

### 2.6. Thermal Gravimetric Analysis (TGA)

TGA analyses were carried out with a MettlerToledo TGA/DSC 1 STARe System equipped with a microbalance. Samples of 8–15 mg were introduced into 100 µL aluminum pans to undergo heating from 20 to 700 °C, with a 10 °C min^−1^ heating rate.

### 2.7. Deferential Scanning Calorimetry (DSC)

DSC results were obtained using a DSC-Q10 calorimeter from TA Instruments. Aluminum sealed crucibles, bearing dried samples of 2–3 mg, were prepared to undergo different heating/cooling cycles, with a heating/cooling rate of 10 °C min^−1^.

### 2.8. Dynamic Rheology

The rheological behavior of the samples in the molten state was studied using an MCR 702 MultiDrive rheometer from Anton Paar. Twenty-five-millimeter-diameter parallel plates were used with a 1 mm gap. The sweeps were performed in the frequency range of 0.1 < ω (rad.s^−1^) < 100 at different temperatures. The strain was set at 2% in order to achieve the experiments in the linear viscoelastic domain which was previously determined for the different temperatures.

### 2.9. Tensile Tests

Tensile tests were achieved using a Zwick 1456 machine. The bulk materials (neat PP and its blend) were dumbbell-shaped for testing; five dumbbells for each one to check the reproducibility. The speed was 20 mm min^−1^. The Young’s modulus, tensile strength and elongation at break were recorded.

The filaments were tested according to ISO 5079 standard. A 10 N sensor was loaded. The distance between the two clamps was set to 20 mm, with a deformation rate of 20 mm/min. Sixty-five monofilaments were tested for each material (neat PP and its blend); their tenacity (cN/Tex) was calculated as the ratio of the tensile strength to the tex. The ambient atmosphere was regulated to ensure standard conditions of temperature (20 ± 2 °C) and relative humidity (65 ± 5%).

### 2.10. Antimicrobial Actsivity

Antimicrobial tests were performed on the obtained textile materials (knits). The antimicrobial activity was determined by transfer method according to ISO 20743 §8.2 (2013). The samples were cut in 3.8 cm-diameter pieces. Each sample was placed on the agar surface of an agar plate that has been previously inoculated (with 1 mL of a bacterial suspension adjusted to be between 1 × 10^6^ and 3 × 10^6^ CFU/mL). A weight of 200 g was applied on the sample for 60 s before placing it in a Petri dish with the transferred surface up. Each sample (as well as each control) was tested in six replicates. Three replicates of each sample (as well as each control) were taken just after the transfer (0 contact time), the bacteria were extracted from them and subjected to counting by using the Plate Count Method. The three other replicates were incubated at 37 °C for 24 h before bacteria extraction and counting (Figure S2 in Supplementary Materials shows the different steps). Two bacterial strains were used: *Staphylococcus aureus* ATCC 6538 and *Klebsiella pneumonia* ATCC 4352.

## 3. Results and Discussion

### 3.1. Casein Hydrolysis

**Figure 1:**
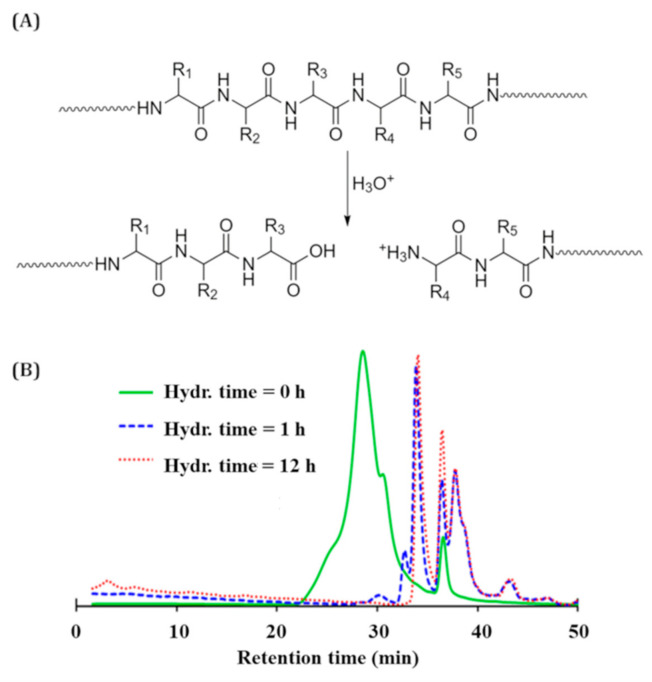
(**A**) Acid hydrolysis mechanism of casein, R_1_, R_2_, R_3_, R_4_ and R_5_ are amino acid groups. (**B**) size exclusion chromatography (SEC) traces (refractive index), in 0.05 mol/L NaCl aqueous solution, for different hydrolysis times.

The acid hydrolysis step led to a decrease in the molecular weight of casein chains; Figure 1 shows the SEC traces corresponding to different hydrolysis times: 0 (before hydrolysis), 1 and 12 h. After 12 h, no evolution of the SEC traces was observed. Figure S3 in Supplementary Materials shows all the obtained traces until 48 h of hydrolysis. The peak of the SEC traces shifts to long retention times, reflecting a decrease in the molecular weight, or in the hydrodynamic radius of casein molecules. This decrease is due to the scission of casein chains [43], by breaking the peptide bonds, according to the chemical mechanism shown in Figure 1A. This reaction is accompanied by the formation of a NH_3_^+^ group that features antibacterial activity [44].

### 3.2. Rheological Study

**Figure 2:**
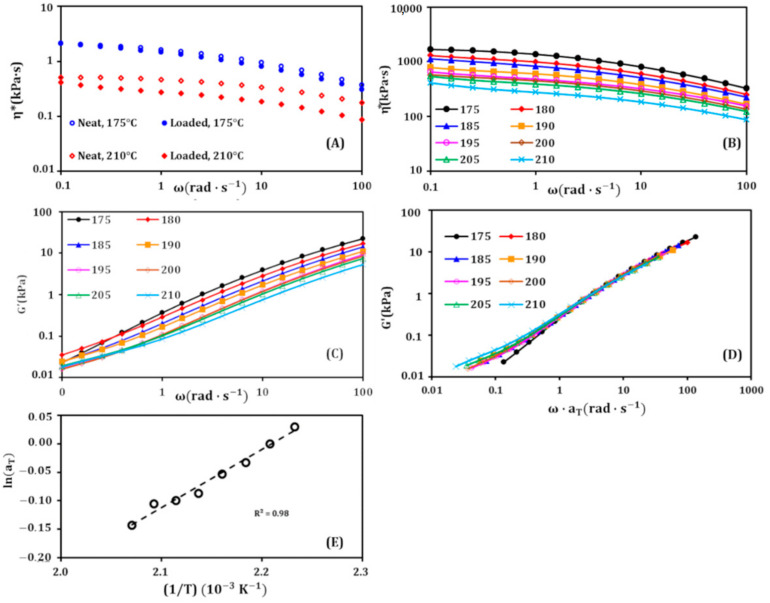
(**A**) Complex viscosity versus angular frequency; comparison between neat polypropylene (PP) (neat) and the PP containing 5 wt.% hydrolyzed casein (loaded), at 175 and 210 °C. (**B**) Complex viscosity versus angular frequency, at different temperatures, for the PP containing 5 wt.% hydrolyzed casein. (**C**) Storage modulus versus angular frequency, at different temperatures, for the PP containing 5 wt.% hydrolyzed casein. (**D**) Superposition of the curves from (**C**) using a_T._ (**E**): ln(a_T_) versus (1/T) allowing the determination of the activation energy E_a_.

To show the effect of the addition of 5 wt.% hydrolyzed casein on the viscosity of the PP matrix, a comparison between the neat and the loaded PP was performed, and Figure 2A shows the result. At 175 °C (the lowest temperature), a small decrease in the viscosity was observed. This decrease becomes more and more important with increasing temperature and also when going to high frequencies, to reach about 50% at 210 °C (at 100 rad.s^−1^). This can be explained by the shear-thinning character of the hydrolyzed casein and its low viscosity compared to PP. Figure 2B illustrates the viscosity curves at different temperatures; it shows that in the investigated range of temperature (175–210 °C) the blend containing 5 wt.% of hydrolyzed casein overall exhibits a shear-thinning behavior with a tendency to form a Newtonian plateau towards low frequencies. By increasing temperature, the Newtonian plateau deforms at low frequencies. This behavior is close to that of neat PP (Figure 2A), except at the leftmost limit of the frequency axis. So, the addition of 5 wt.% hydrolyzed casein decreases the viscosity of the PP considerably, and changes its rheological behavior only at low frequencies.

Figure 2C shows the curves of the storage modulus (G′) obtained by achieving frequency sweeps at different temperatures. It can be seen the G′ value decreases by increasing temperature. The shape of the curves is the same in the most part of the frequency range, except at the low frequencies (same observation as for viscosity). This behavior is very similar to that described in the literature [45,46] as a thermorheologically complex behavior. Since the hydrolyzed casein chains bear ionic pendant groups (NH_3_^+^), physical (coulombic) interactions may occur between them and affect the relaxation mechanisms of the polymer [47,48,49]. According to Tobolsky et al. [50], the pendant ionic groups find themselves in an environment which is thermodynamically unfavorable to dissolve them: they are surrounded by a hydrocarbon polymeric medium. Therefore, the chains with pendant ionic groups tend to aggregate thanks to the coulombic interactions, thus disturbing the relaxation processes.

To further investigate this behavior, a superposition of the G′ curves was achieved (Figure 2D). As predicted above, the curves are easily superimposable in the major part of the frequency range, with some offsets towards low frequencies. The superposition was carried out by using a temperature shift factor $a_{T}$ defined by:

(1) $$a_{T}=\frac{\eta \left(T\right)}{\eta \left(T_{0}\right)}$$

where $\eta \left(T\right)$ and $\eta \left(T_{0}\right)$ are, respectively, the viscosities at the temperature T and the reference temperature T_0_, which is 175 °C in this study. When the angular frequency ω is multiplied by this factor, the G′ curves are horizontally shifted to be superimposed on each other. The values of a_T_ are found to be dependent on temperature following the Arrhenius equation [51,52,53]:

(2) $$a_{T}={\mathrm{Ae}}^{\frac{E_{a}}{\mathrm{RT}}}$$

where A is a pre-exponential factor, E_a_ the activation energy of the phenomenon, R the universal gas constant and T the temperature. There is no minus sign in the left of E_a_ because this energy is taken as the energy opposed to the relaxation process (a barrier to the relaxation).

The linearization of the previous equation allows us to write:

(3) $$\ln\left(a_{T}\right)=\frac{E_{a}}{\mathrm{RT}}+\ln\left(A\right)$$

By plotting $\ln\left(a_{T}\right)$ as a function of $\frac{1}{T}$, a linear relation was found (R^2^ = 0.98), as shown in Figure 2E. This result confirms the Arrhenius behavior and allows the determination of E_a_ from the slope of the line; it is found to be equal to 90.1 kJ mol^−1^. This value is in accordance with those found in the literature and corresponding to such interactions (ionic weak interactions) [54,55,56].

Neat PP was also subjected to the same study (Figure S4 in Supplementary Materials), and the activation energy of the relaxation process was found to be 59.8 kJ mol^−1^, lower than the E_a_ of the blend. This increase in E_a_ after adding the hydrolyzed casein (ΔE_a_ = 30.3 kJ mol^−1^) can be attributed to the interactions between NH_3_^+^ groups of hydrolyzed casein chains, that hinder the relaxation of the polymer chains. It can also be attributed to the formation of crystalline regions of hydrolyzed casein chains (aggregates or ensembles) favored by the thermodynamic aspects discussed above.

### 3.3. Thermal Study

**Figure 3:**
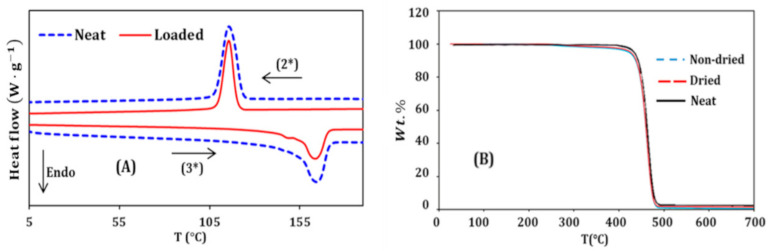
(**A**) DSC curves of the neat PP (Neat) and the blend containing 5 wt.% hydrolyzed casein (Loaded). (2*): cooling ramp. (3*): second heating ramp. (**B**) TGA curves of the blend.

Differential scanning calorimetry measurements were achieved on both the neat PP and the blend with 5 wt.% hydrolyzed casein. Each sample underwent a first heating ramp from 0 to 200 °C, before the cooling (2*) and heating (3*) ramps shown in Figure 3A. No shifting was observed for the crystallization peak; it still remains at 117 °C after adding the hydrolyzed casein. But the enthalpy of crystallization, obtained by integrating the peaks, decreased from 94.3 to 63.8 J g^−1^. This decrease means that the crystallization phenomenon is lessened, probably because of the interactions between NH_3_^+^ groups that hinder the polymer chain motion, and inhibit their migration to the growing crystals [57]. This result is in accordance with the energy activation calculations achieved in the previous part.

The endothermal melting peak at 163.8 °C was also not shifted, but it shows a small significant shoulder on its left side after the addition of hydrolyzed casein. This can be related to the previous result regarding the crystallization peak; since the kinetics the formation of nuclei and the growth of the crystals are affected by adding hydrolyzed casein, the semi-crystalline structure of the PP may be changed, and thus the melting (relaxation) process occurs differently from one region to other (at the scale of the crystals) within the blend [58]. This interpretation is also supported by the fact that this small shoulder is not observable during the first heating ramp (Figure S5 in Supplementary Materials). Many authors have encountered this phenomenon [59,60].

**Figure 4:**
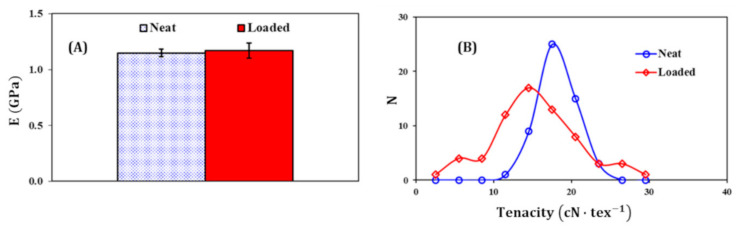
(**A**) Young’s modulus of neat PP and filled one, tests achieved on dumbbell-shaped samples. (**B**) Number distribution of the tenacity in the two populations of filaments: made of either neat or filled PP.

In the field of textiles, the moisture content of the materials and their hygroscopy are very important parameters [61,62]. In order to assess the moisture content of the PP/hydrolyzed casein blend, thermogravimetric analyses were performed. Two samples were used: the first one was kept in the ambient atmosphere for 3 weeks, and the second was dried under vacuum, at 60 °C, for 24 h. The obtained TGA curves are shown in Figure 4; it can be seen that the two curves are almost identical. The analysis of the recorded values (tables) allowed the determination of the weight percentage lost at different temperatures: at 110 °C, which is higher than the boiling point of water, no loss (0.00 wt.%) was found for the two samples. At 120 °C, no loss (0.00 wt.%) was found in the case of the dried sample, and only a 0.04 wt.% loss was calculated for the non-dried one. It can be concluded that there is almost no moisture in the blend, even when kept in ambient conditions. For comparison, cotton fibers contain between 5 and 10 wt.% of moisture [63] and wool-based textiles can reach 40 wt.% [64]. The TGA curve of the neat PP is also illustrated in Figure 3B, and it shows slight differences compared to the loaded PP curves: at 300 °C, the neat PP loses 0.3 wt.%—this loss was equal to 1 and 1.2 wt.%, respectively, for the dried and non-dried samples containing hydrolyzed casein. At 400 °C, neat PP loses 0.4% of its weight, while dried and non-dried blends lost, respectively, 2.1 and 2.9 wt.%. The inflection point (maximum degradation rate) occurs at nearly the same temperature: 121 °C for the neat PP, and 119 °C for the two loaded samples.

### 3.4. Mechanical Properties

The Young’s modulus values from tensile tests are illustrated in Figure 4A for dumbbells made of either neat PP or 5 wt.% hydrolyzed casein loaded one (stress-strain curves are in Supplementary Materials, Figure S6). The difference between the two values is within the margin of error, as shown by the error bars: an average value of 1.15 ± 0.03 GPa was found for neat PP and 1.19 ± 0.07 GPa for the filled one. It can be concluded that the stiffness of the PP was not significantly affected by addition of 5 wt.% hydrolyzed casein. The other parameters (strength and strain) are also slightly affected; they are summarized in Figure S7 (Supplementary Materials).

Figure 4B shows the number distribution of the tenacity values obtained by testing a population of 65 filaments of PP containing 5 wt.% hydrolyzed casein and the same number of filaments made of neat PP. The bell curve of the filled PP is broader than that of neat PP; this indicates a better homogeneity in the case of neat PP filaments from point of vu of tenacity. This figure also illustrates that the addition of 5 wt.% hydrolyzed casein shifts the distribution curve (and its top) to lower tenacities. This reflects a decrease in the tensile strengths of the filled filaments. The observed weakening, and also the disparity of the tenacity values of the blend, can be attributed to the poor adhesion of PP matrix with hydrolyzed casein, causing structural defects [65]: PP is hydrophobic, in contrast to NH_3_^+^ groups of hydrolyzed casein, which are hydrophilic. To fix this constraint, a compatibilizer (such as amphiphilic polymer or compound) can be used to enhance the adhesion between the hydrophobic matrix and the hydrolyzed casein chains [66,67]. According to the literature, maleic anhydride-grafted polypropylene seems to be an effective candidate [68,69].

### 3.5. Antibacterial Activity

Antibacterial activity tests were achieved on the knit made of PP containing 5 wt.% hydrolyzed casein, and also on a knit made of neat PP as control sample. Two bacterial strains were used: *Staphylococcus aureus* (Gram+) and *Klebsiella pneumoniae* (Gram−). The results are summarized in Table 3

For the two bacterial strains, the presence of the neat PP (control) sample on the agar surface did not prevent the bacterial growth: the number of bacterial colony forming units (CFU) increases from 10^4^ to 10^7^ after 24 h of incubation. The growth value F, which is defined as the variation of log(CFU)between t = 0 and t = 24 h, is positive and equal to 2.5 and 2.6 for *Staphylococcus aureus* and *Klebsiella pneumoniae*, respectively.

When the loaded sample is used (knit made of PP containing 5 wt.% hydrolyzed casein), the CFU number decreases from 1.6 × 10^5^ to 0, and from 1.4 × 10^4^ to 0 for the Gram+ and Gram− strains, respectively. This indicates not only an impediment of the bacterial growth, but also a total elimination of the bacteria after 24 h incubation. Thus, the growth value G (defined as the variation of log(CFU) between t = 0 and t = 24 h for the active sample) is negative in these two cases (resp. < −3.9 and < −3.8).

According to the ISO 20743 (2013) norm, the antibacterial efficacy can be quantified by using the antibacterial activity value A, defined by A=F−G, and by following the classification mentioned in Table 4. The values of A, in this work, were found to be >6.4 for the two bacterial strains. This value indicates a very strong antibacterial activity, due to the addition of 5 wt.% hydrolyzed casein in the PP matrix.

**Figure 5:**
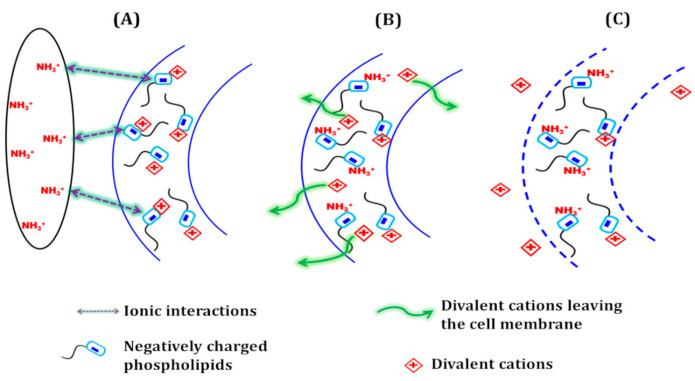
Schematic representation of the antibacterial action of ammonium pendent groups from hydrolyzed casein. (**A**) Electrostatic interactions between the material and the bacterial cell membrane. (**B**) Association of ammonium groups with negatively charged phospholipids in the membrane. (**C**) Deterioration of the membrane.

The effective antibacterial action of the blend elaborated here can be explained by strong electrostatic interactions between the hydrolyzed casein chains and the bacterial cell membranes, since the cationic pendant ammonium group (NH_3_^+^) in hydrolyzed casein is positively charged, and the cell membranes of the bacteria contains negatively charged phospholipids [70,71,72,73,74,75]. These interactions represent a first and crucial step of the antibacterial mechanism, before killing the cell. Figure 5 shows the different steps of the supposed mechanism: the negative charges of the phospholipids are naturally equilibrated by divalent cations such as calcium (Ca^2+^) and magnesium (Mg^2+^). The first step (A) consists of mutual attractions between the cell membrane and positively charged pendent groups from the hydrolyzed casein chains. Once these groups are in contact with the membrane, the negatively charged groups of the phospholipids interact better with NH_3_^+^ than with their natural counter-ions (step B). As a consequence, the divalent cations are released (step C) and the cell membrane loses its integrity, causing cell death.

## 4. Conclusions

Filaments were successfully made from a blend of PP with 5 wt.% hydrolyzed casein, allowing the elaboration of yarns and a knitting fabric. The latter showed high efficiency as an antibacterial material; its antibacterial activity value was found to be higher than 6.4. For comparison, silver and ZnO are widely used as antibacterial agents in textile materials; recently developed silver-based textiles showed an antibacterial activity value of 4.5 against *Staphylococcus aureus* [76]. Another new textile material based on cotton covered with ZnO nanoparticles was designed; it showed antibacterial activity values ranging from 1.2 to 4.0 towards five different bacterial strains, Gram+ and Gram−, among which we find *Staphylococcus aureus* [77].

The rheological study leads us to suppose physical interactions between NH_3_^+^ groups of hydrolyzed casein; these interactions hinder the relaxation process of the blend but do not significantly affect the rheological behavior of PP. Viscosity, at a high temperature, was however decreased by the addition of 5 wt.% hydrolyzed casein. This difference has to be considered during a possible process at the industrial scale.

The difference in the hydrophilic/hydrophobic character between the matrix (hydrocarbon chains of PP) and the hydrolyzed casein chains bearing NH_3_^+^ groups seemed to slightly affect the mechanical properties of the PP; the effect was clearly visible only for the filaments. Further future works may fix this issue by adding a suitable compatibilizer to the blend.

No moisture retention was observed for the obtained knit compared to neat PP; this is a valued property in the field of textile materials.

In addition to the interesting features mentioned above, the textile material was made in a melt process (extrusion and melt spinning) following simple steps; this represents a relevant advantage for the current environmental context in which environmentally friendly processes are highly desired.

These results confirm that it is possible to replace some currently used antibacterial agents that are not natural, non-renewable, and harmful to the environment as well as to the human health, with a product from a renewable source, while promoting the recycling of dairy waste.

## Figures and Tables

**Table 1:** Temperatures in the different zones of the extruder.

Extrusion Temperature Profile					
Zone	Z_1_ (feed)	Z_2_	Z_3_	Z_4_	Z_5_ (die)
T (°C)	160	170	180	190	200

**Table 2:** Parameters of the spinning process.

Temperature Profile in the Single-Screw Extruder						Parameters of the Rolls				
						Roll 1		Roll 2		R
z_1_	z_2_	z_3_	z_4_	z_5_	z_6_	T (°C)	S_1_ (rpm)	T (°C)	S_2_ (rpm)	2.5
175	180	190	190	195	195	70	80	80	200	

**Table 3:** Results of antibacterial activity tests.

			0 Contact Time	After 24 h
**Test towards *Staphylococcus aureus***	Neat PP	Trial 1	4.4 × 04	1.3 × 107
		Trial 2	4.4 × 104	9.2 × 106
		Average CFU	4.4 × 104	1.1 × 107
		Log(average CFU)	4.9	7.1
		Growth value F	2.5	
	5 wt.% loaded	Trial 1	1.8 × 105	<20 *
		Trial 2	1.3 × 105	<20 *
		Average CFU	1.6 × 105	<20
		Log(average CFU)	5.2	<1.3
		Growth value G	<−3.9	
	A=F−G		>6.4	
**Tests towards *Klebsiella pneumoniae***	Neat PP	Trial 1	4.6 × 104	4.0 × 107
		Trial 2	1.1 × 105	2.8 × 107
		Average CFU	7.8 × 104	3.4 × 107
		Log(average CFU)	4.9	7.5
		Growth value F	2.6	
	5 wt.% loaded	Trial 1	2.2 × 105	<20 *
		Trial 2	5.0 × 104	<20 *
		Average CFU	1.4 × 105	<20
		Log(average CFU)	5.1	<1.3
		Growth value G	<−3.8	
	A=F−G		>6.4	

* No colony detected, has to be written as <20 because the count is performed on 1 mL coming from a wash solution of 20 mL.

**Table 4:** Classification of antibacterial efficacy.

Efficacy of Antibacterial Property	Antibacterial Value A
Significant	2 ≤ A < 3
Strong	A ≥ 3

## Data Availability

No new data were created or analyzed in this study. Data sharing is not applicable to this article.

## Supplementary Materials

The following are available online at https://www.mdpi.com/1996-1944/14/2/251/s1, Figure S1: (a): The yarn obtained by melt spinning of PP containing 5wt.% hydrolyzed casein. (b): The knit made of the yarn, Figure S2: Schematic representation of the followed steps during the antibacterial tests, according to the ISO 20743 §8.2 (2013) norm, Figure S3: SEC traces casein at different times of hydrolysis, Figure S4: Neat PP rheological study. (A): Storage modulus at different temperatures. (B): Superposition of G′ using a thermal shift factor aT. (C): Ea determination from the plot of ln(aT) versus (1/T), Figure S5: DSC curves corresponding to the first heating ramp, for neat PP and 5wt.% loaded one, Figure S6: Stress-strain curves, from tensile tests, for the neat PP (five runs) and the PP loaded with 5wt.% hydrolyzed casein (five runs), Figure S7. Strain (ε) and stress (σ) values from tensile tests achieved on dumbbells of neat PP (Neat) or 5wt.% hydrolyzed casein loaded PP (Loaded).
