# Supplementary material for: Antibacterial Textile Based on Hydrolyzed Milk Casein
Source: Materials (Basel). 2021 Jan 6;14(2):251. doi: 10.3390/ma14020251 (PMC7825562; doi:10.3390/ma14020251)
Supplement: Supplementary file 1 [file materials-14-00251-s001.pdf]

materials

*Supplementary Material*

# Antibacterial Textile Based on Hydrolyzed Milk Casein

**Kedafi Belkhir [1], Caroline Pillon [2,3,4], Aurélie Cayla [1] and Christine Campagne [1,*]**

[1] ENSAIT, GEMTEX—Laboratoire de Génie et Matériaux Textiles, F-59000 Lille, France; kedafi.belkhir@centralelille.fr (K.B.); aurelie.cayla@ensait.fr (A.C.)
[2] Université de Lyon, F-42023 Saint Etienne, France; caroline.pillon@univ-st-etienne.fr
[3] CNRS, UMR5223, Ingénierie des Matériaux Polymères, F-42023 Saint-Etienne, France
[4] Université Jean Monnet de Saint Etienne, F-42023 Saint-Etienne, France
* Correspondence: christine.campagne@ensait.fr

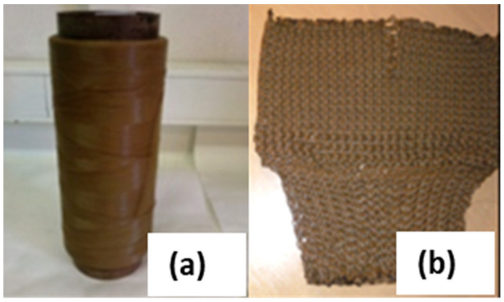


**Figure S1.** (**a**) The yarn obtained by melt spinning of PP containing 5 wt.% hydrolyzed casein. (**b**) The knit made of the yarn.

**Citation:** Belkhir, K.; Pillon, C.; Cayla, A.; Campagne, C. Antibacterial Textile based on Hydrolyzed Milk Casein. *Materials* **2021**, *14*, 251. https://doi.org/10.3390/ma14020251

Received: 18 December 2020
Accepted: 4 January 2021
Published: 6 January 2021

**Publisher's Note:** MDPI stays neutral with regard to jurisdictional claims in published maps and institutional affiliations.

**Copyright:** © 2021 by the authors. Licensee MDPI, Basel, Switzerland. This article is an open access article distributed under the terms and conditions of the Creative Commons Attribution (CC BY) license (http://creativecommons.org/licenses/by/4.0/).

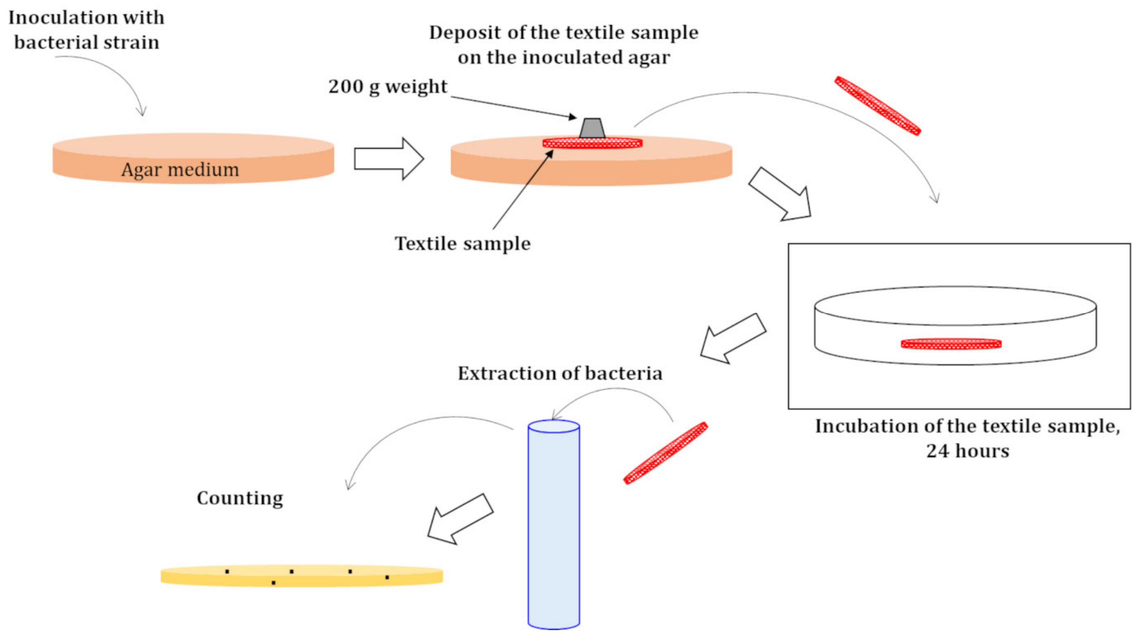


**Figure S2.** Schematic representation of the followed steps during the antibacterial tests, according to the ISO 20743 §8.2 (2013) norm.

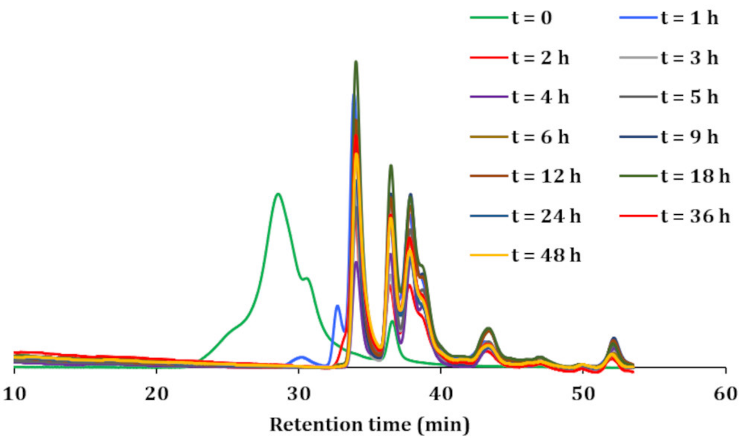


**Figure S3.** SEC traces casein at different times of hydrolysis.

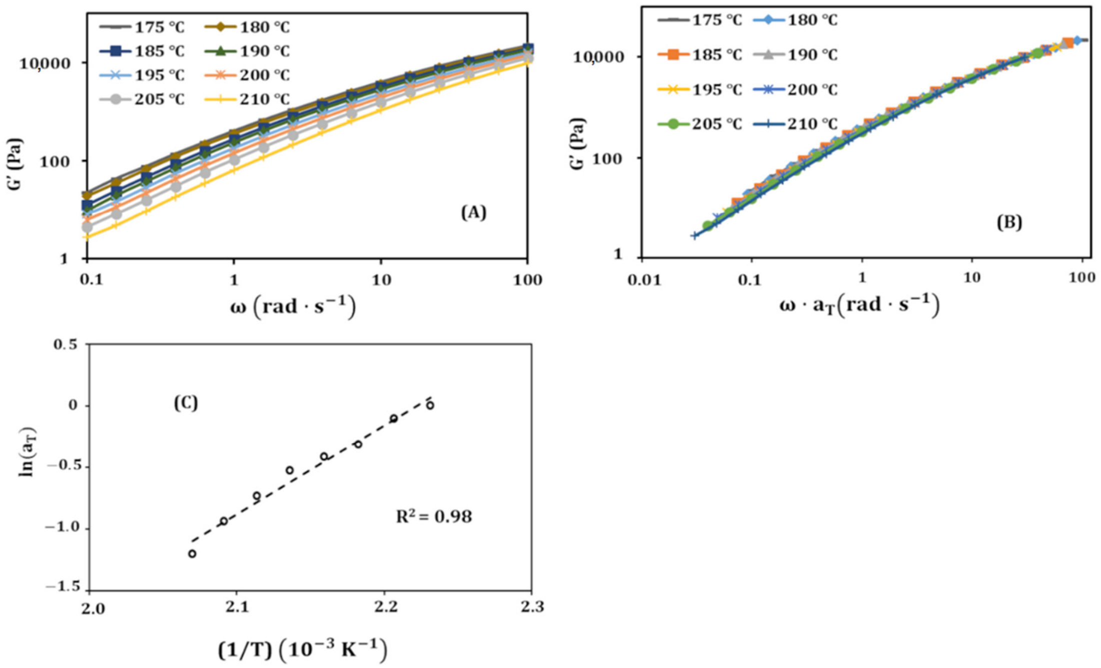


**Figure S4.** Neat PP rheological study. (**A**) Storage modulus at different temperatures. (**B**) Superposition of G′ using a thermal shift factor aT. (**C**) $E_a$ determination from the plot of $\ln(a_T)$ versus (1/T).

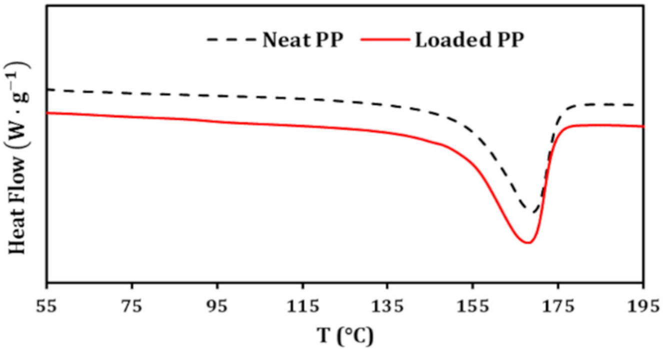


**Figure S5.** DSC curves corresponding to the first heating ramp, for neat PP and 5 wt.% loaded one.

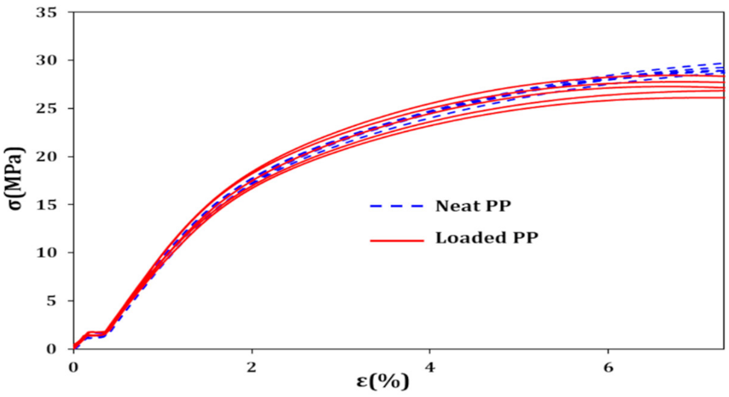

**Figure S6.** Stress-strain curves, from tensile tests, for the neat PP (five runs) and the PP loaded with 5wt.% hydrolyzed casein (five runs).

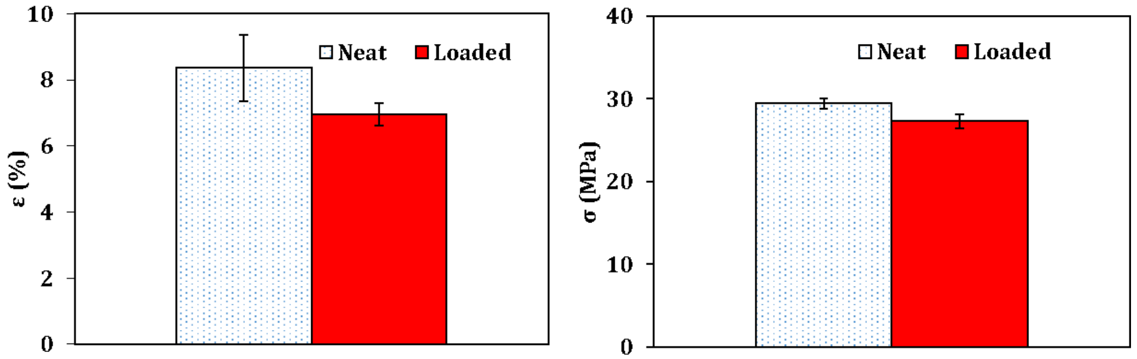


**Figure S7.** Strain (ε) and stress (σ) values from tensile tests achieved on dumbbells of neat PP (Neat) or 5 wt.% hydrolyzed casein loaded PP (Loaded).
